# Protecting the Lungs

**Published:** 1875-10

**Authors:** 


### PROTECTING THE LUNGS.

It is very much the fashion now-a-days for persons to put on some sort of a chest-protector, of cloth or buckskin, as cold weather advances. Those are rendered necessary on the part of men, by the habit of wearing coats and vests cut low, so as to leave very little except the two thicknesses comprised in the shirt-bosom and the undershirt, to cover and clothe the region of the lungs. That these chest-pads serve to ward off the piercing blasts of winter, and to render the individual less liable to pneumonia is evident. No substance is better adapted to this use than the skin of the sheep or goat, as it is not easily penetrated from without. The plan of perforating the skins used in underclothes and pads, is useless and injurious, as the very object—that of excluding air—is thereby annulled.

There are buckskin appliances—pads and belts—devised by Dr. Cooper, of Boston, which serve an admirable purpose as protectors, and have a still greater advantage, in the fact that between the folds is stitched some suitable medicines, to act upon the skin and to be taken up by the absorbents. These patent medicated pads have been used for a year or two to some extent, and are reported to have relieved some severe cases of bronchitis and rheumatism. We know of one case in which a lady was, as she testifies, greatly benefited by wearing one of the belts about the waist. She was greatly reduced in flesh when she began wearing the belt, but she began to gain directly, and soon grew strong and rosy, adding 20 lbs. to her weight in three months.

These pads and belts are sold in New York, at the drug-stores of Hegeman & Co., and possibly by others. We are free to say, that, from all we know of their reputed value, we should wear them in preference to any others, and should anticipate good results, beyond such as would attend mere protection of the parts.

---

### A PRESENT FOR THE BOYS.

The season of seasons for gifts—the holiday time—is approaching, and our friends are casting about in their minds as to what good and useful thing shall be given to the children. In "hard times" like these, it is far better to give the useful than the ornamental. There are a number of articles which we have thought of recommending as presents, some costing little and others costing more, and perhaps we will speak of some of them in November or December. Just now we will only allude to one—and that is Mr. Joseph Watson's little Printing-Offices, sold by him at his two stores, No. 53 Murray Street, New York, and No. 73 Cornhill, Boston.

Here now, is a gift for a boy, which, if he has any desire to be a man, to do and be anything, will gladden his heart. It may cost all the way up from two dollars, for even this small sum will buy the smallest press. But whatever the size, and style, and price, it will be good and serviceable, and will produce good work. We have tried these presses, and can testify to their strength and to the excellence of the impression which they give. We can print cards and circulars, and letter-heads and bill-heads, and larger jobs even, as well as we could on the most costly press in the world.

Boys, and even girls, like to set type and to print, and the work so nearly approaches literary labor that it becomes a grand educator. Besides, boys and girls can earn a good deal of money by the help of one of these little presses, and that is not a thing to be despised.

Now we will say to any boy who buys one of Mr. Watson's presses, and will send us a fair specimen of his work, accompanied with a letter telling us how he gets along with the press and types, that we will publish his letter, and if he has any difficulty, we will tell him how to avoid it in the future.

